# Orderly Regulation of Macrophages and Fibroblasts by Axl in Bleomycin‐Induced Pulmonary Fibrosis in Mice

**DOI:** 10.1111/jcmm.70321

**Published:** 2025-01-08

**Authors:** Xinyu Zhao, Yupeng Li, Shengnan Yang, Yicong Chen, Kaiwei Wu, Jing Geng, Peipei Liu, Zai Wang, Huaping Dai, Chen Wang

**Affiliations:** ^1^ The Second Affiliated Hospital of Harbin Medical University Heilongjiang China; ^2^ Department of Pulmonary and Critical Care Medicine, Center of Respiratory Medicine, National Clinical Research Center for Respiratory Diseases China‐Japan Friendship Hospital Beijing China; ^3^ National Center for Respiratory Medicine, Institute of Respiratory Medicine Chinese Academy of Medical Sciences Beijing China; ^4^ Department of Respiratory and Critical Care Medicine Tianjin Chest Hospital China; ^5^ Capital Medical University Beijing China; ^6^ Peking Union Medical College Beijing China; ^7^ Department of Medicine and Women's Guild Lung Institute Cedars‐Sinai Medical Center Los Angeles California USA; ^8^ Institute of Clinical Medical Sciences China‐Japan Friendship Hospital Beijing China

**Keywords:** Axl, fibroblast, lung injury, macrophage, pulmonary fibrosis

## Abstract

Pulmonary fibrosis is a pathological manifestation that occurs upon lung injury and subsequence aberrant repair with poor prognosis. However, current treatment is limited and does not distinguish different disease stages. Here, we aimed to study the differential functions of Axl, a receptor tyrosine kinase expressing on both macrophages and fibroblasts, in the whole course of pulmonary fibrosis. We used mice with Axl total knockout, conditionally knockout in macrophages or fibroblasts, or treating with Axl inhibitors in inflammation or fibrosis stages to examine the effect of temporary dysfunction of Axl on bleomycin (BLM)‐induced pulmonary fibrosis. Primary bone marrow–derived monocytes and primary fibroblasts from mice were used for cell‐type–specific studies. Lung tissue and plasma samples were collected from idiopathic pulmonary fibrosis (IPF) patients and healthy controls to assess the Axl levels. We found that Axl inhibited the M1 polarisation of macrophages; inhibition of Axl during acute phase exacerbated inflammatory response and subsequent pulmonary fibrosis. On the other hand, Axl promoted the proliferation and invasion of the fibroblasts, partially by accelerating the focal adhesion turnover; inhibiting Axl during the fibrotic phase significantly alleviated pulmonary fibrosis. Consistently, phosphorylated Axl levels increased in fibrotic foci in the lung sample of IPF patients. In contrast, the soluble Axl (sAxl) level decreased in their plasma as compared to healthy controls. These results indicate that Axl may sequentially and differentially regulate macrophages and fibroblasts in acute and fibrosis phases, implying the necessity of a stage‐specific treatment for pulmonary fibrosis. In addition, the activated Axl on fibroblasts may be reflected by the lowered plasma sAxl level, which may act as a biomarker for IPF.

**Trial Registration:** ClinicalTrials.gov identifier: NCT03730337

**Abbreviations**
AMs: alveolar macrophages
ARDS: acute respiratory distress syndrome
BLM: bleomycin
Col‐1: collagen Type I
FBS: foetal bovine serum
Gas 6: growth arrest‐specific 6
HYP: hydroxyproline
IFN‐γ: immune interferon
IL: interleukin
IMs: interstitial macrophages
IPF: idiopathic pulmonary fibrosis
M‐CSF: macrophage colony‐stimulating factor
MoMs: monocyte‐derived macrophages
PBS: phosphate buffer saline
sAxl: soluble Axl
TGF‐β: transforming growth factor‐β
TNF‐α: tumour necrosis factor‐α
WB: western blot
α‐SMA: α‐smooth muscle actin

## 1 Introduction

Pulmonary fibrosis is a pathological manifestation caused by excessive extracellular matrix and collagen deposition during the aberrant repair process following lung injury [1]. Idiopathic pulmonary fibrosis (IPF) is a chronic, progressive disease of the lungs. It is a type of pulmonary fibrosis and has the worst prognosis, with a median survival of only 2–3 years [2, 3, 4]. However, current treatment is limited. Treatment with pirfenidone and nintedanib can only improve lung function, but cannot reduce mortality [5]. Therefore, it is still necessary to explore new targets for pulmonary fibrosis treatment.

The occurrence of IPF begins with repeated subtle damage of alveolar epithelial cells, followed by fibrosis due to abnormal repair. Its pathogenesis can be roughly divided into two stages: acute inflammatory phase and subsequent fibrosis phase. Additionally, some patients experience an acute exacerbation due to infection or other injury, which can result in a worse prognosis [6]. However, current therapeutic interventions do not involve the differentiation of the different disease stages. In particular, the optimal treatment for acute exacerbations in patients with IPF is still uncertain [7], which may limit the accuracy and efficacy of existing therapies.

There is increasing evidence that macrophages and fibroblasts are key cell types that affect pulmonary fibrosis [2, 8]. Macrophages may play an important role in acute inflammation. During lung injury, tissue‐resident macrophages (TRMs), including alveolar macrophages (AMs) and interstitial macrophages (IMs), are consumed. Circulating monocyte‐derived macrophages (MoMs) are recruited to lung tissues and participate in inflammation and repair processes [9, 10]. It is worth noting that higher peripheral blood mononuclear cell counts are associated with poorer prognosis in IPF patients [11], emphasising the importance of monocytes and macrophages in the pathology of IPF. These findings suggest that both TRMs and MoMs may contribute significantly to the inflammatory response of IPF. In the fibrosis stage, fibroblasts can differentiate into myofibroblasts after activation and acquire the ability to proliferate, resist apoptosis, invade, migrate and secrete an excessive extracellular matrix, which plays a major role in the formation of fibrosis [12].

Axl is a cell surface receptor tyrosine kinase (RTK) that belongs to the TAM (Tyro3, Axl and Mer) kinase family along with Mer and Tyro3. Axl is highly expressed in macrophages [13, 14, 15]. Activated by its ligand Gas6, it can play a role in negatively regulating the inflammatory response of macrophages through PI3K/Akt/NF‐κB, PI3K/Akt/Bcl‐2, STAT3 and other pathways. However, the function of Axl pathway in the macrophages during the inflammatory phase of IPF is unknown. Interestingly, studies in cancer suggest that Axl can promote the invasion and migration of various tumour cells. In the study of pulmonary fibrosis, it was found that the Axl‐targeting inhibitor R428 can effectively inhibit the proliferation and migration of pulmonary fibroblasts in patients with IPF, as well as inhibit pulmonary fibrosis in humanised SCID/Bg mice [16], suggesting that targeting Axl and its downstream signalling pathways may be a new therapeutic strategy in the fibrosis stage.

Therefore, it is intriguing whether Axl plays a different role by acting on different cell types in different stages of IPF. In this study, Axl inhibitors, Axl total or conditional knockout mice were used to study the orderly regulation of macrophages and fibroblasts by Axl in mice models of lung fibrosis induced by bleomycin (BLM), to start an initial exploration of precise treatment strategies for different stages of IPF.

## 2 Results

### 2.1 Total Inhibition or Knockout of Axl Does Not Reduce BLM‐Induced Pulmonary Fibrosis in Mice

**FIGURE 1:**
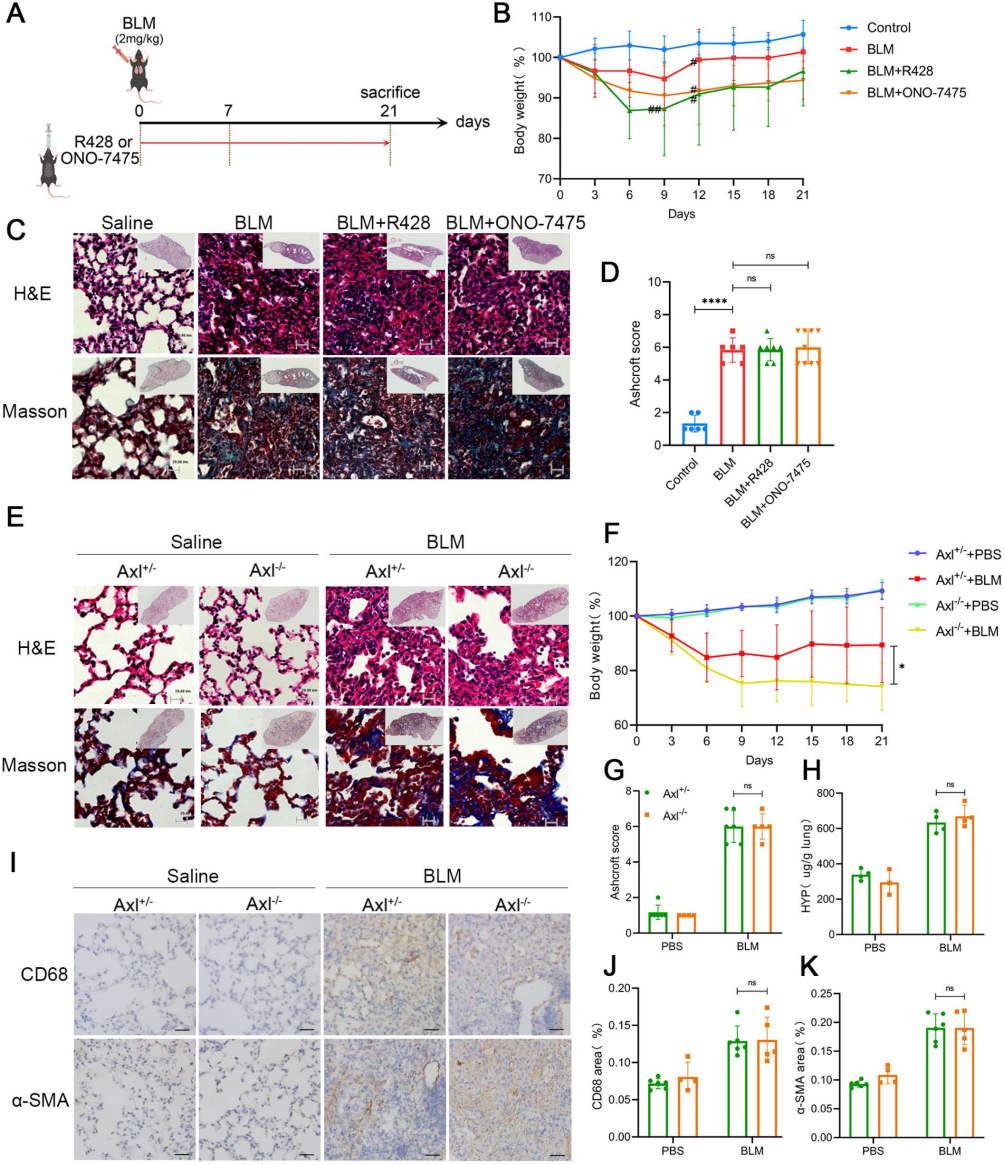
BLM modelling in mice with Axl total inhibition or knockout. (A) After BLM treatment, mice were administered R428 or ONO‐7475 from Day 0 to Day 21. (B) Body weight curves of the aforementioned mice. Initially, control group (*n* = 6), BLM group (*n* = 7), R428 group (*n* = 10) and ONO‐7475 group (*n* = 10). Three mice in the R428 group, one in the ONO‐7475 group and one in the BLM group perished during the administration of BLM. ^#^ represents the death of one mouse. (C) H&E staining and Masson staining after sampling on Day 21. Scale bars: All 20 μm. Control group (*n* = 6), BLM group (*n* = 6), R428 group (*n* = 7) and ONO‐7475 group (*n* = 9). (D) Ashcroft scores of mice in Figure C. (E) Axl^−/−^ or Axl^+/−^ mice treated with BLM were sampled on Day 21 for H&E staining and Masson staining. Scale bars: All 20 μm. (F) The weight curves of the mice in Figure E. (G) Ashcroft scores of mice in Figure E. (H) Hydroxyproline contents in lung tissues of mice in Figure E. (I) Immunohistochemistry of paraffin sections of mice's lung tissues in Figure E. Scale bars: All 50 μm. (J, K) Immunohistochemical quantification of CD68 (J) and α‐SMA (K) protein levels in Figure I. *n* = 4–10 mice/group, ****p < 0.0001. # represents the death of a mouse. ## means two dead mice.

We first studied the effects of Axl inhibition on pulmonary fibrosis using an animal model. Axl inhibitors bemcentinib (R428) and ONO‐7475 (Figure 1A) were injected intragastrically into mice from Day 0 to Day 21 after BLM treatment. R428 is a small‐molecule inhibitor that highly selectively inhibits Axl via ATP competition. ONO‐7475 also targets Axl and Mer through competitive inhibition, thereby inhibiting the biological functions of both. The body weight curve (Figure 1B), haematoxylin and eosin (H&E) staining, Masson staining (Figure 1C) and the Ashcroft score (Figure 1D) all indicated that there was no relief of pulmonary fibrosis in mice in the R428 and ONO‐747 groups, while the body weight curve of mice in the two groups decreased more significantly than that of the BLM group.

We then used Axl knockout mice (KO) to further verify the effect of Axl deletion on pulmonary fibrosis. BLM administration experiments were conducted on Axl^−/−^ mice and their littermate control Axl^+/−^ mice. The results of H&E staining, Masson staining (Figure 1E) and the Ashcroft score (Figure 1G) were similar to those of the mice treated with the Axl inhibitor. The absence of Axl did not reduce pulmonary fibrosis in mice. Furthermore, Axl^−/−^ mice showed more severe weight loss (Figure 1F). Hydroxyproline (HYP) was measured in the lung tissue to accurately determine the level of pulmonary fibrosis. The results showed that there was no significant difference in the pulmonary fibrosis level between Axl^−/−^ and Axl^+/−^ mice (Figure 1H). Immunohistochemistry (IHC) of the lung tissues showed that the expression of CD68 and alpha‐smooth muscle actin (α‐SMA) were increased after BLM administration, and there was also no difference in expression between Axl^−/−^ and Axl^+/−^ mice (Figure 1I–K). These results demonstrated that total inhibition or absence of Axl did not reduce BLM‐induced pulmonary fibrosis in mice.

### 2.2 Inhibition of Axl During the Acute Inflammatory Stage Aggravates BLM‐Induced Pulmonary Fibrosis

**FIGURE 2:**
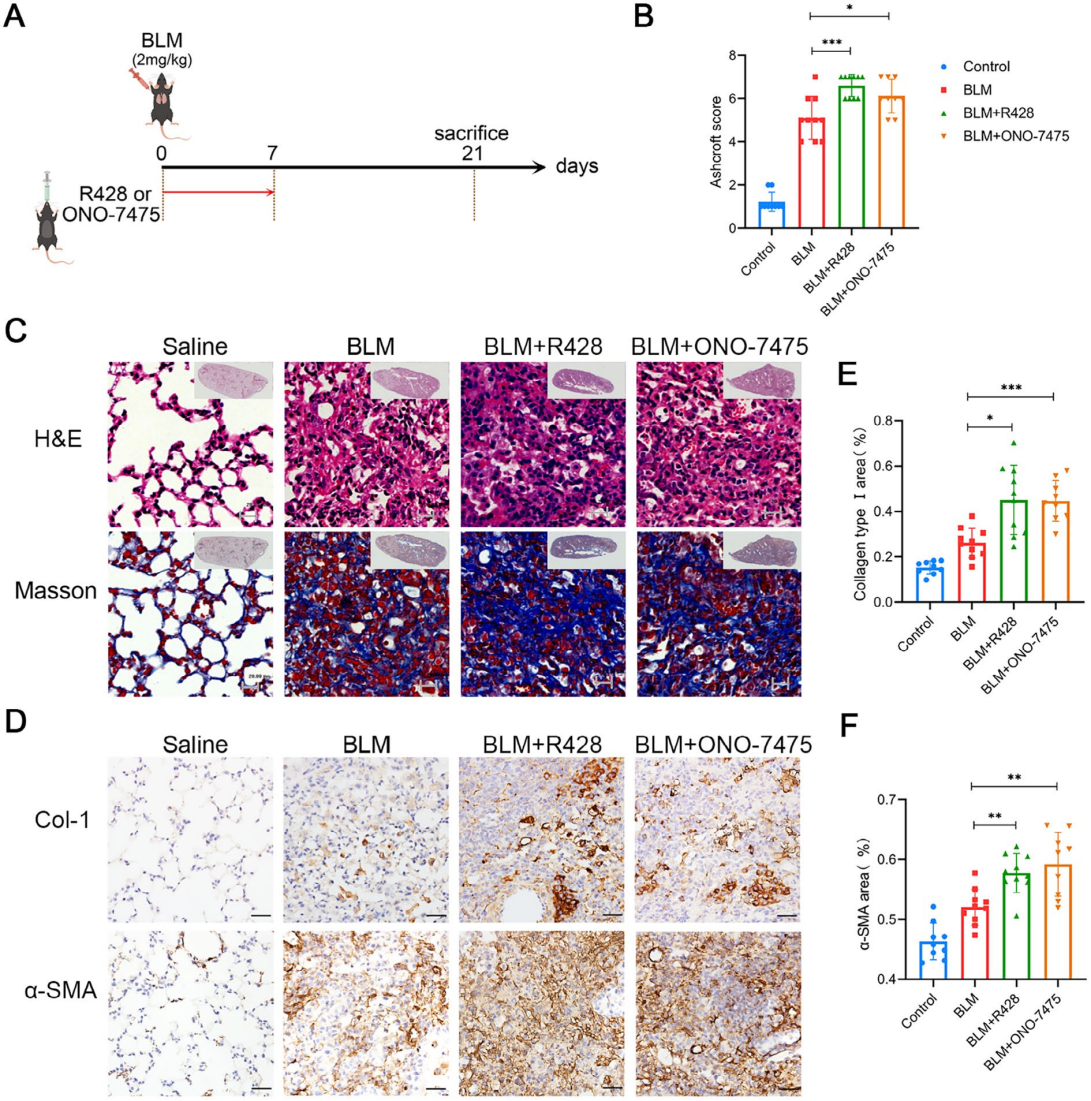
Inhibition of Axl in the acute phase of pulmonary fibrosis. (A) After BLM administration, mice were given R428 and ONO‐7475, respectively, and the administration time was from Day 0 to Day 7. (B‐C) Ashcroft scores (B), H&E staining and Masson staining (C) after sampling on Day 21. Scale bars: All 20 μm. (D) Protein levels of collagen Type I and α‐SMA were determined by immunohistochemistry. Scale bars: All 50 μm. (E, F) Immunohistochemical quantification of collagen Type I (E) and α‐SMA (F) protein levels in mice in Figure D. *n* = 9–10 mice/group, */**/***p < 0.05/0.01/0.001.

Since whole course inhibition of Axl did not achieve an effective therapeutic result, we further examined the function of Axl in different stages. The mouse model was divided into acute inflammatory stage (Day 0 to Day 7) and the fibrosis stage (Day 8 to Day 21). We first studied the effects of Axl inhibitors applied from Day 0 to Day 7 on the fibrosis of the lungs excised at Day 21 (Figure 2A). According to H&E, Masson staining (Figure 2C) and the Ashcroft score (Figure 2B), lung tissue sections from the inhibitor groups contained more interstitial cells and collagen deposition. IHC showed increased levels of collagen Type I (Col‐1) and α‐SMA (Figure 2D–F). Thus, inhibiting Axl activity at the acute inflammatory response stage can lead to proliferation of myofibroblasts and increased collagen deposition in the subsequent period, thus aggravating the progression of fibrosis.

### 2.3 Axl Inhibition During Acute Inflammatory Stage Exacerbates BLM‐Induced Lung Injury

Since Axl exerts anti‐inflammatory properties by regulating macrophage activity [17], we speculated that inhibition of Axl in the acute inflammatory period could induce a more serious inflammatory response and lung injury by regulating macrophage activity, which may lead to the excessive recruitment of mononuclear/macrophages from the blood to lung tissue and promote fibrosis.

**FIGURE 3:**
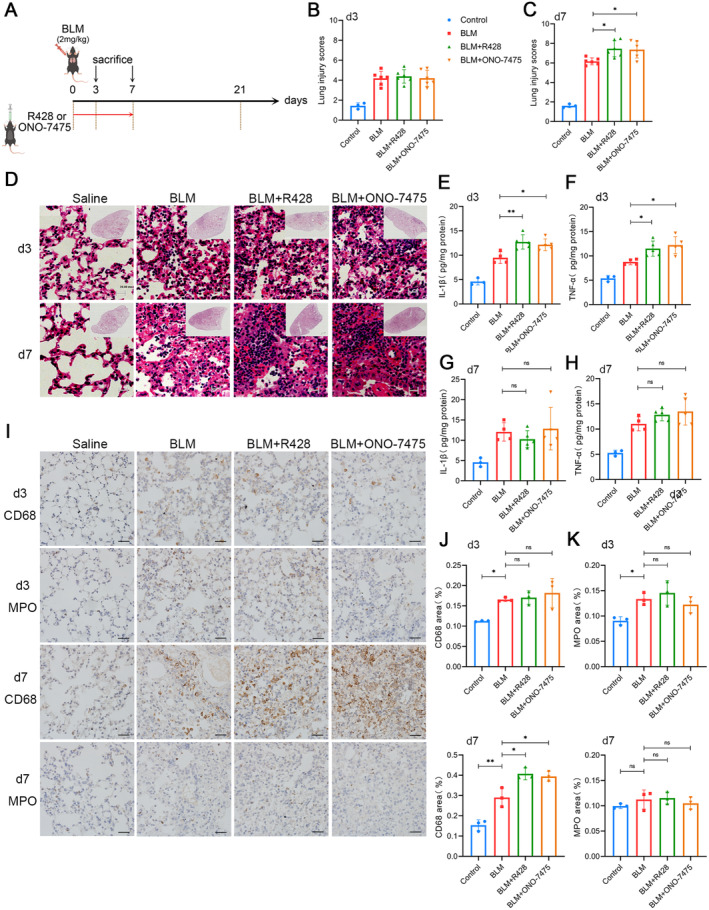
Axl inhibition at the stage of acute inflammatory reaction can aggravate lung injury. (A) After BLM treatment, mice were given R428 or ONO‐7475 and the administration time was from Day 0 to Day 3/7 by intragastric administration, and samples were collected on Day 3/7. (B–D) H&E staining and lung injury scores were performed after sampling on Day 3 or 7. Scale bars: All 20 μm. (E–H) The expression levels of IL‐1β and TNF‐α were detected by ELISA after grinding mice's lung tissues on Day 3 or 7. (I) Immunohistochemistry of mice's lung tissues on Day 3 or 7. Scale bars: All 50 μm. (J–M) Protein levels of CD68 (J) and MPO (K) in lung tissues on Day 3 and CD68 (L) and MPO (M) in lung tissues on Day 7 of immunohistochemical quantification in mice in Figure I. *n* = 3–6 mice/group, */**p < 0.05/0.01.

To verify this hypothesis, we first treated mice with inhibitors in the inflammation stage and harvested the lungs on Day 3 or Day 7 (Figure 3A). We found that BLM caused alveolar exudation on Day 3 after administration. H&E staining and the lung injury score analysis showed that the degree of lung injury of the inhibitor‐treated groups and the BLM group was similar. However, on Day 7 after BLM administration, the alveolar exudation and the inflammatory cell infiltration increased, and the lung injury aggravated in the R428 or ONO‐7475 treatment groups compared with the BLM control group (Figure 3B–D). ELISA using lung tissue samples showed that the inflammatory factors IL‐1β and TNF‐α were higher in the inhibitor groups than those in the BLM group on Day 3 (Figure 3E,F), but the difference was not obvious on Day 7 (Figure 3G,H). At the same time, immunohistochemical staining was performed on the lung tissues of mice (Figure 3I), which showed that there was no significant difference in the expression of CD68 between the BLM group and inhibitor groups on Day 3, but CD68 levels in the inhibitor groups on Day 7 were higher than that in the BLM group (Figure 3J,L). On Days 3 and 7, MPO staining of excised lung tissue showed no significant differences in the recruitment of neutrophils between the inhibitor groups and the BLM group.

We also performed a transcriptome sequencing analysis of lung tissues with different treatments. Changes in inflammatory response and clotting‐related pathways were found in mice in the inhibitor groups and the BLM group (Figure S1A,B). On Day 3 and Day 7, the scores of NF‐κB, PI3K/Akt and STAT‐related pathways in the inhibitor groups were significantly higher than those of the BLM group. For the inflammatory factors Il‐1b, Il‐6 and Tnf, although there was no statistical difference between the inhibitor groups and the BLM group, they showed a trend of increased levels in the inhibitor groups on Day 3 (Figure S1C–E). On the contrary, the transcription levels of these inflammatory factors showed a declining trend in the inhibitor groups on Day 7 (Figure S1F–H), suggesting that the Axl inhibition in the acute inflammatory phase may promote a rapid and stronger activation of inflammatory pathways.

### 2.4 Axl Regulates Macrophage Polarisation

In the acute stage of lung injury, the proinflammatory M1 macrophages dominate, which may aggravate the tissue injury. During the tissue repair phase, MoMs tend to polarise into profibrotic M2 macrophages, leading to the activation of fibroblasts and uncontrolled fibrotic repair [10].

**FIGURE 4:**
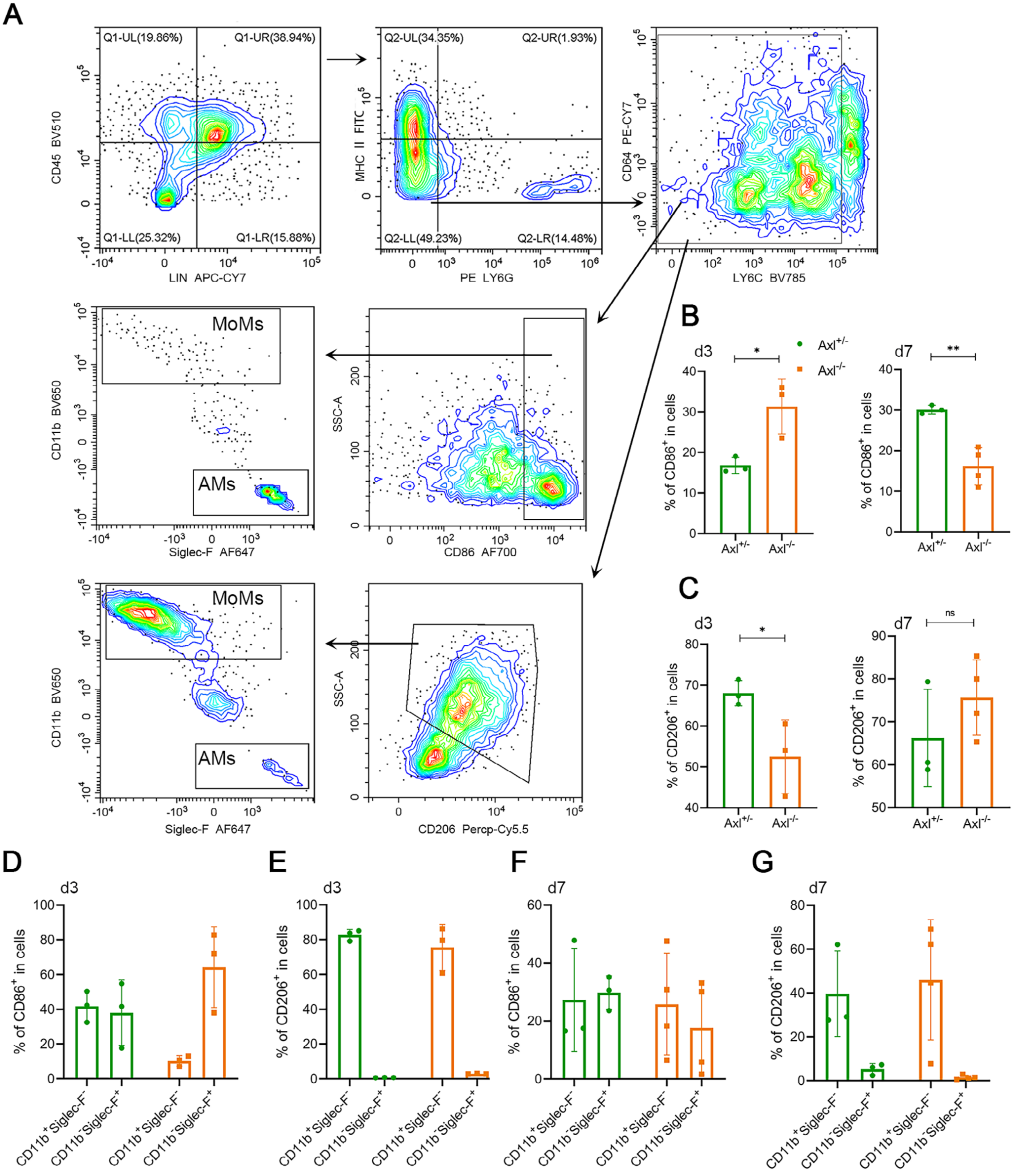
Flow cytometry of Axl knockout mice's lung tissues after BLM administration. (A) On Day 3 or Day 7 after BLM administration, single‐cell suspensions of lung tissues were prepared and analysed by flow cytometry. (B) The figure on the left and the figure on the right show the proportion of M1 macrophages in the macrophage population on the 3rd and 7th days, respectively. (C) The figure on the left and the figure on the right show the proportion of M2 macrophages in the macrophage population on the 3rd and 7th days, respectively. (D, E) The percentage of AMs and MoMs in M1 (D) and M2 (E) macrophages sampled from lung tissues on Day 3. (F, G) The percentage of AMs and MoMs in M1 (F) and M2 (G) macrophages sampled from lung tissues on Day 7. *n* = 3 mice/group, */**p < 0.05/0.01.

To investigate the regulatory effect of Axl on macrophage polarisation, we utilised lung samples from Axl^−/−^ and Axl^+/−^ mice 3 or 7 days after BLM administration. Lung tissue samples were processed into a single‐cell suspension and were labelled for a series of cell surface markers to identify M1 and M2 macrophages. We used CD86 and CD206 as cell surface markers for M1 and M2, respectively [18]. MoMs and AMs were identified according to CD11b and Siglec‐F expression. CD11b^+^Siglec‐F^−^ identifies MoMs, and CD11b^−^Siglec‐F^+^ identifies AMs (Figure 4A). The number of M1 macrophages was higher in Axl^−/−^ mice than in Axl^+/−^ mice on Day 3 (Figure 4B, left), whereas it showed an opposite result on Day 7 (Figure 4B, right), indicating a rapid proinflammatory response of macrophages upon Axl knockout. Meanwhile, the number of M2 macrophages was significantly lower than that in Axl^+/−^ mice on Day 3 (Figure 4C, left) and showed no significant difference on Day 7, although the trend was opposite compared with Day 3 (Figure 4C, right).

To trace the origin of M1 and M2 macrophages, we further analysed M1 and M2 macrophages based on AM or MoM markers. The results showed that M1 macrophages in lung tissue were mainly composed of AMs rather than MoMs in Axl^−/−^ mice, while it was composed of approximately equal amounts of AMs and MoMs in Axl^+/−^ mice on Day 3. This difference was not present on Day 7. M2 macrophages were mainly composed of MoMs in both Axl^−/−^ and Axl^+/−^ mice on both Day 3 and Day 7 (Figure 4D–G). Combined with the results of inhibitor administration in the acute phase, these data suggest that the difference in macrophage polarisation caused by Axl deficiency may occur earlier than pathological changes during the inflammatory response stage. The inhibition of Axl resulted in an increase in M1 macrophage from AM on Day 3, which may be a reason for the worsening of lung injury on Day 7.

**FIGURE 5:**
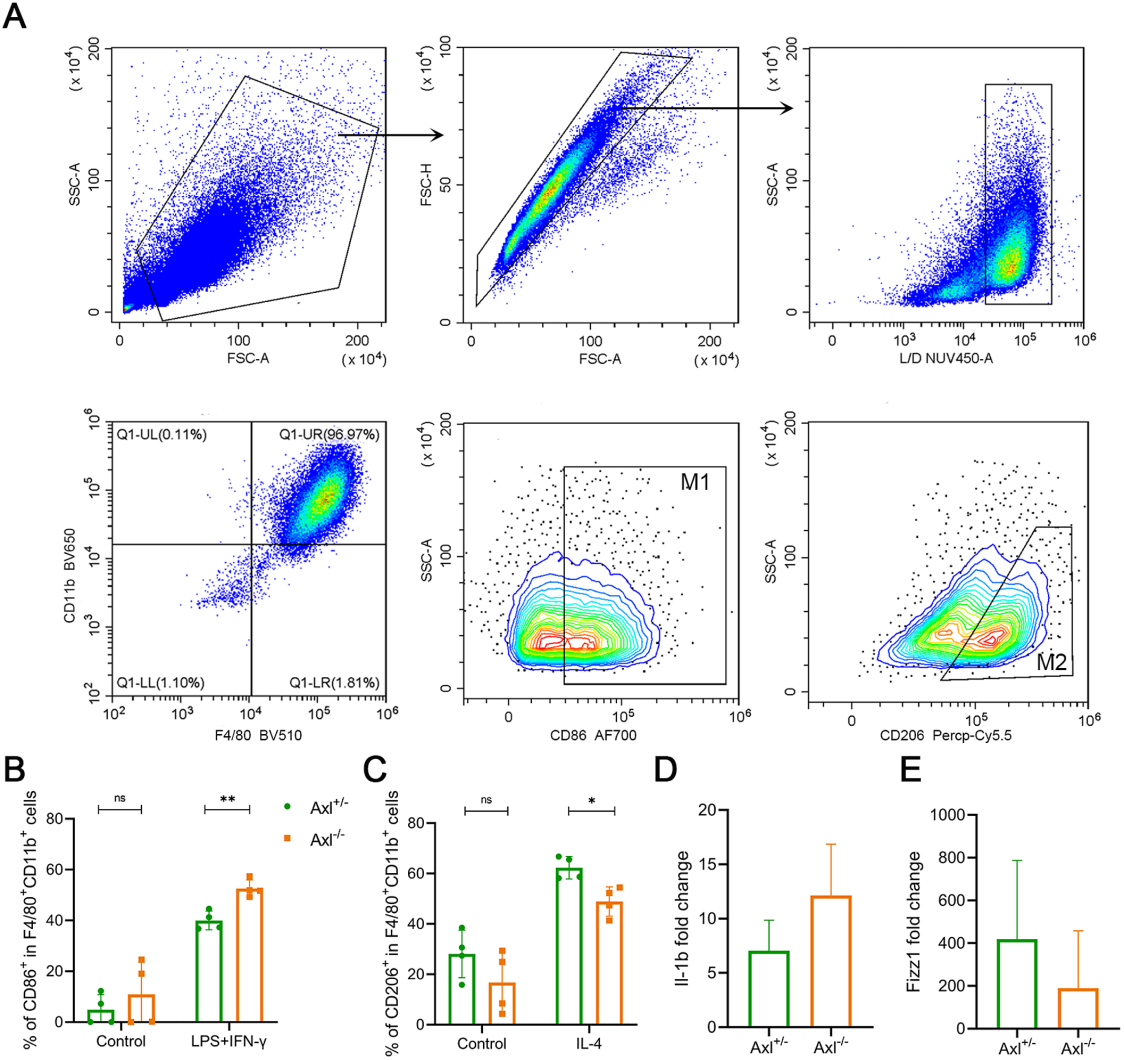
Axl regulated the polarisation of BMDM. (A) Flow cytometry analysis. (B) The proportion of M1 macrophages in the mononuclear/macrophage population. (C) The proportion of M2 macrophages in mononuclear/macrophage population. (D, E) RNA was extracted from the cells and the expressions of Il‐1b (D) and Fizz1 (E) were detected by real‐time PCR. *n* = 3–4 mice/group, */**p < 0.05/0.01.

To further investigate the Axl deficiency on macrophage polarisation, we extracted bone marrow–derived macrophages (BMDMs) from Axl^−/−^ and Axl^+/−^ mice, and treated them with LPS plus IFN‐γ for M1 polarisation, or IL‐4 for M2 polarisation. Flow cytometry was used to analyse the ratio of M1 and M2 in Axl^−/−^ and Axl^+/−^ cells. We defined F4/80^+^CD11b^+^ as mature macrophages and used CD86 and CD206 as cell surface markers of M1 and M2, respectively (Figure 5A). We found that the proportion of induced M1 macrophages was higher in Axl^−/−^ cells than in Axl^+/−^ cells (Figure 5B), while the proportion of induced M2 macrophages showed an opposite result (Figure 5C). To further verify the effect of Axl knockout on macrophage polarisation, we used real‐time RT‐PCR to determine the expression of Il‐1b, a marker of M1 macrophages, and Fizz1, a marker of M2 macrophages. Due to the large variation between individual mice, we did not obtain statistical differences between groups. However, there was a trend that the expression level of Il‐1b was higher in Axl^−/−^ cells than in Axl^+/−^ cells, while Fizz1 expression showed an opposite result (Figure 5D,E). These data suggested that macrophages may be more likely to polarise into M1 macrophages upon Axl knockout. At the same time, Axl may also inhibit the M2 polarisation of macrophages.

### 2.5 Axl Plays Different Roles in Lung Macrophages and Fibroblasts

Due to the diminishment of tissue‐resident AMs after BLM administration, the AM pool was quickly supplemented by mainly MoMs. To investigate the function of Axl in MoMs, we constructed Cx3cr1‐Cre::Axl^fl/fl^ mice and examined the formation of pulmonary fibrosis after BLM administration. Additionally, since Axl was also expressed in fibroblast and may promote their activation, we used Col1a2‐Cre::Axl^fl/fl^ mice to confirm the effects of Axl deletion in fibroblast and myofibroblasts on BLM‐induced pulmonary fibrosis.

**FIGURE 6:**
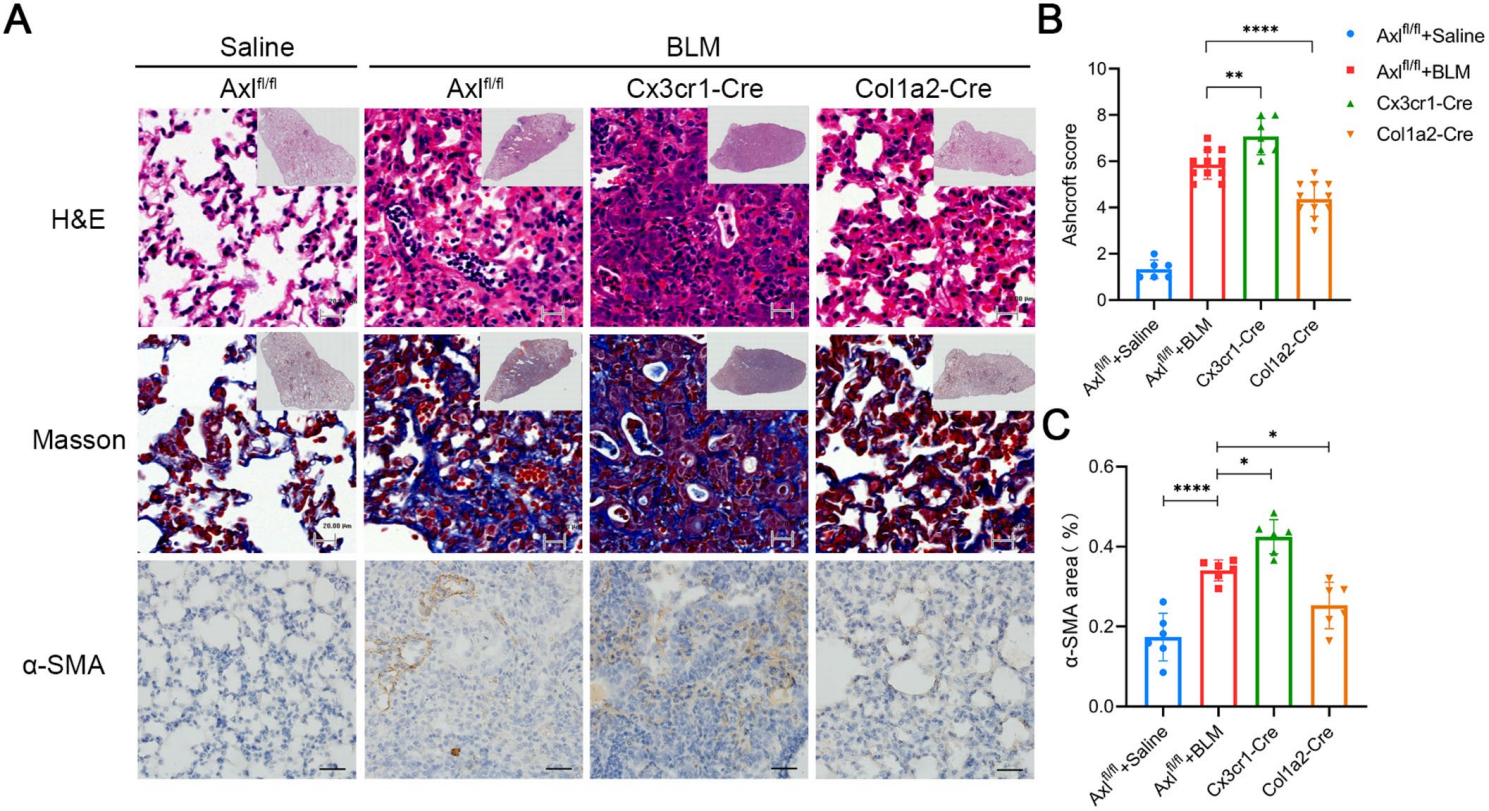
Axl acts differently in macrophages and fibroblasts. (A) On Day 21 after BLM modelling, mice's lung tissues were selected for H&E staining, Masson staining (scale bars: all 20 μm) and α‐SMA immunohistochemical staining (scale bars: all 50 μm). (B) Ashcroft scores of the above mice. (C) Immunohistochemical quantitative levels of α‐SMA. *n* = 6–11 mice/group, */**/****p < 0.05/0.01/0.0001.

As a result, H&E staining, Masson staining and the Ashcroft score all indicated that Axl specifically knockout in MoMs could aggravate pulmonary fibrosis, while Axl specifically knockout in fibroblasts could alleviate pulmonary fibrosis on Day 21 after BLM administration in mice (Figure 6A,B). Immunohistochemical staining of lung tissue showed that α‐SMA expression was higher in the Cx3cr1‐Cre group, but lower in the Col1a2‐Cre group when compared to the BLM control group (Figure 6A,C). These results further indicate that Axl plays different roles in inhibiting or promoting fibrosis in macrophages and fibroblasts, respectively.

### 2.6 Inhibition of Axl During the Fibroplasia and Fibrosis Phase Reduces Pulmonary Fibrosis

**FIGURE 7:**
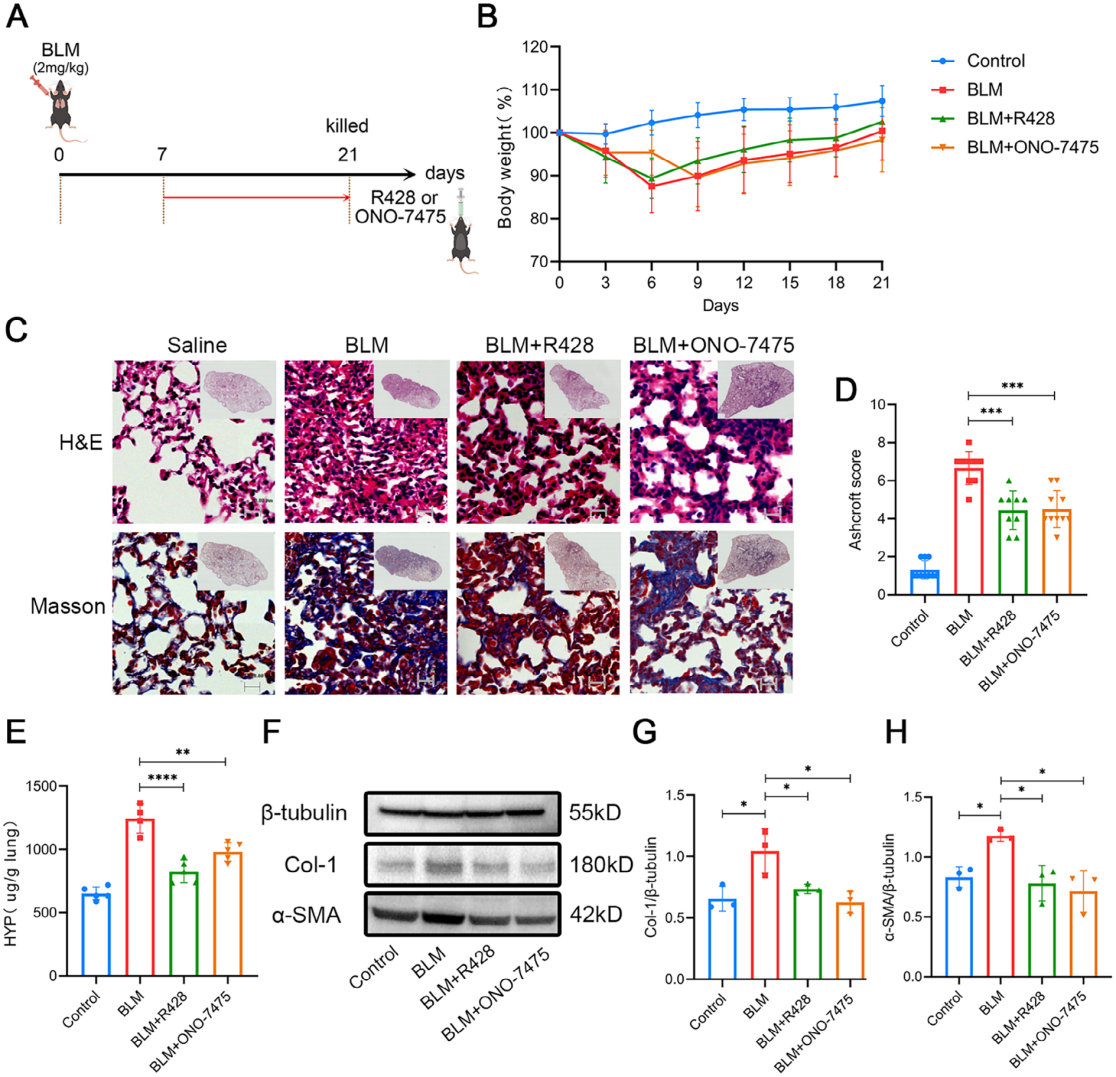
Inhibiting Axl during the fibrotic phase reduced pulmonary fibrosis. (A) After BLM treatment, mice were given R428 or ONO‐7475, and the administration time was from Day 7 to Day 21. (B) Body weight curves of mice within 21 days. (C) H&E staining and Masson staining were performed after paraffin sections of the above mice's lung tissues. Scale bars: All 20 μm. (D) Ashcroft scores of mice in Figure C. (E) Hydroxyproline quantifications in lung tissues on Day 21. (F) Lung tissues were lysed and WB was performed, Collagen Type I and α‐SMA results were obtained. Three samples in each group were mixed and repeated three times. (G, H) Quantitative results of collagen Type I (G) and α‐SMA (H) in Figure F. *n* = 3–10 mice/group. */**/***/****p < 0.05/0.01/0.001/0.0001.

To further verify the results of Col1a2‐Cre::Axl^fl/fl^ model mice and confirm that Axl promotes fibroblast activation, we conducted experiments using Axl inhibitors in the fibroplasia and fibrotic phase (Day 7 to Day 21, referred to as the fibrosis phase). Mice were given R428 or ONO‐7475 by gavage from Day 7 to Day 21 after BLM administration and lung tissues were collected on Day 21 (Figure 7A). Mice in the R428 group regained weight faster than those in the BLM and ONO‐7475 groups during the fibrosis phase (Figure 7B). H&E, Masson staining and the Ashcroft score showed that the degree of pulmonary fibrosis in the two inhibitor groups was lower than that in the BLM group alone (Figure 7C,D). The HYP content in the inhibitor groups was significantly lower than that in the BLM group (Figure 7E). Western blotting analysis also demonstrated that the protein levels of Col‐1 and α‐SMA in the lung tissues of the inhibitor groups were significantly lower than those of the BLM group (Figure 7F,G). The above results indicate that inhibiting Axl during the fibrosis phase can alleviate the formation of pulmonary fibrosis.

### 2.7 Axl Deficiency Inhibits Fibroblast Proliferation and Migration

**FIGURE 8:**
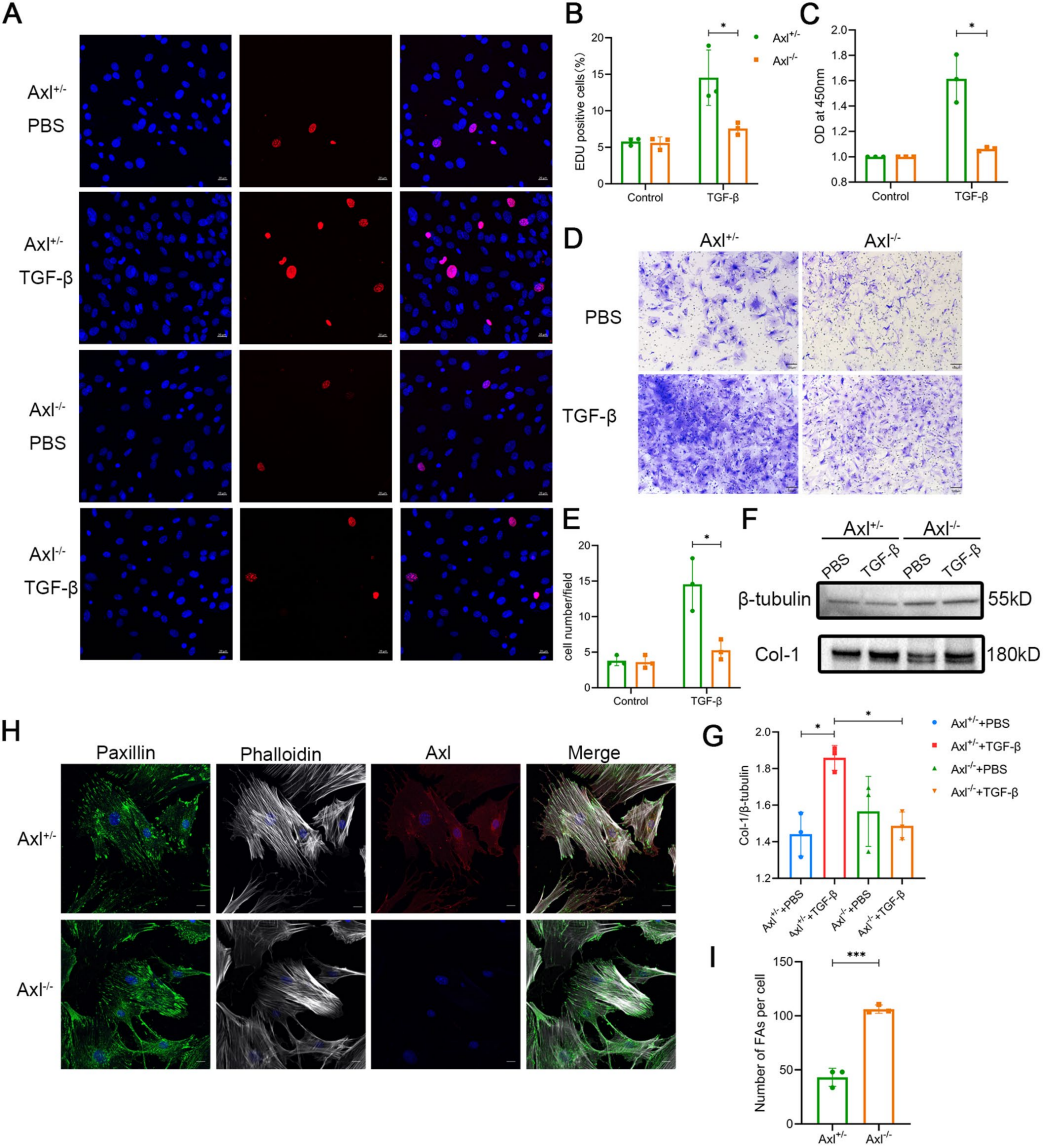
Axl deficiency could inhibit the proliferation and migration of fibroblasts. The following experiments were performed after primary fibroblasts were treated with TGF‐β or PBS for 48 h. (A) EDU incubation for 2 h and immunofluorescence detection. Scale bars: All 20 μm. (B) Quantitative results in Figure A. (C) The results of cell proliferation were detected by CCK‐8. (D) Photograph of the cell invasion experiment described above. Scale bars: All 100 μm. (E) The invasion experiment of the above cells was quantitative, and five random visual fields were selected from each Transwell under ×400 magnification for statistics. (F) Cell proteins were extracted and the protein levels of collagen Type I were assessed by western blotting. (G) Quantitative results in Figure F. (H) The cells were stained by immunofluorescence with PXN, phalloidin and Axl, and then photographed under confocal microscopy. Scale bars: All 20 μm. (I) PXN was quantified in Figure H. A total of 15 cells were selected from each slide under ×400 magnification for quantification. *n* = 3 mice/group, */***p < 0.05/0.001.

Although R428 has been reported to significantly inhibit the proliferation, migration and synthetic activity of fibroblasts derived from IPF patients [16], there has been no genetic manipulation research to validate the role of Axl in fibroblasts. We extracted and cultured Axl^−/−^ and Axl^+/−^ mouse primary fibroblasts to the third‐to‐fifth generations for follow‐up experiments. We stimulated fibroblasts to differentiate into myofibroblasts using TGF‐β and then measured cell proliferation using the EDU assay. The results showed that the number of cells in the proliferative stage was significantly lower in the Axl knockout group (Figure 8A,B). The CCK‐8 assay also verified that Axl depletion could inhibit cell proliferation (Figure 8C). Cell invasion experiments demonstrated that deletion of Axl inhibited the invasion ability of myofibroblasts after being stimulated by TGF‐β (Figure 8D,E). Western blotting further demonstrated that Axl knockout could reduce the expression of Col‐1 in myofibroblasts (Figure 8F,G). These data confirmed the role of Axl in promoting aggressive phenotypes in fibroblasts/myofibroblasts.

Axl may regulate cell proliferation through the Akt/Erk pathway, but how Axl regulates myofibroblast invasion and migration is less clear. Referring to the findings in tumour research [19], we speculated that Axl may affect the activity of actin‐binding proteins to accelerate the turnover of focal adhesion (FA), promote the elimination of the original anchor sites with the matrix and generate new anchor sites after cell movement. Paxillin (PXN) was used to mark the FA of myofibroblasts. Immunofluorescence staining of PXN showed that the number of FA in Axl^−/−^ cells was significantly higher than that in Axl^+/−^ cells (Figure 8H,I), indicating that Axl may facilitate FA turnover, which may promote the migration of myofibroblasts and fibrosis development.

**FIGURE 9:**
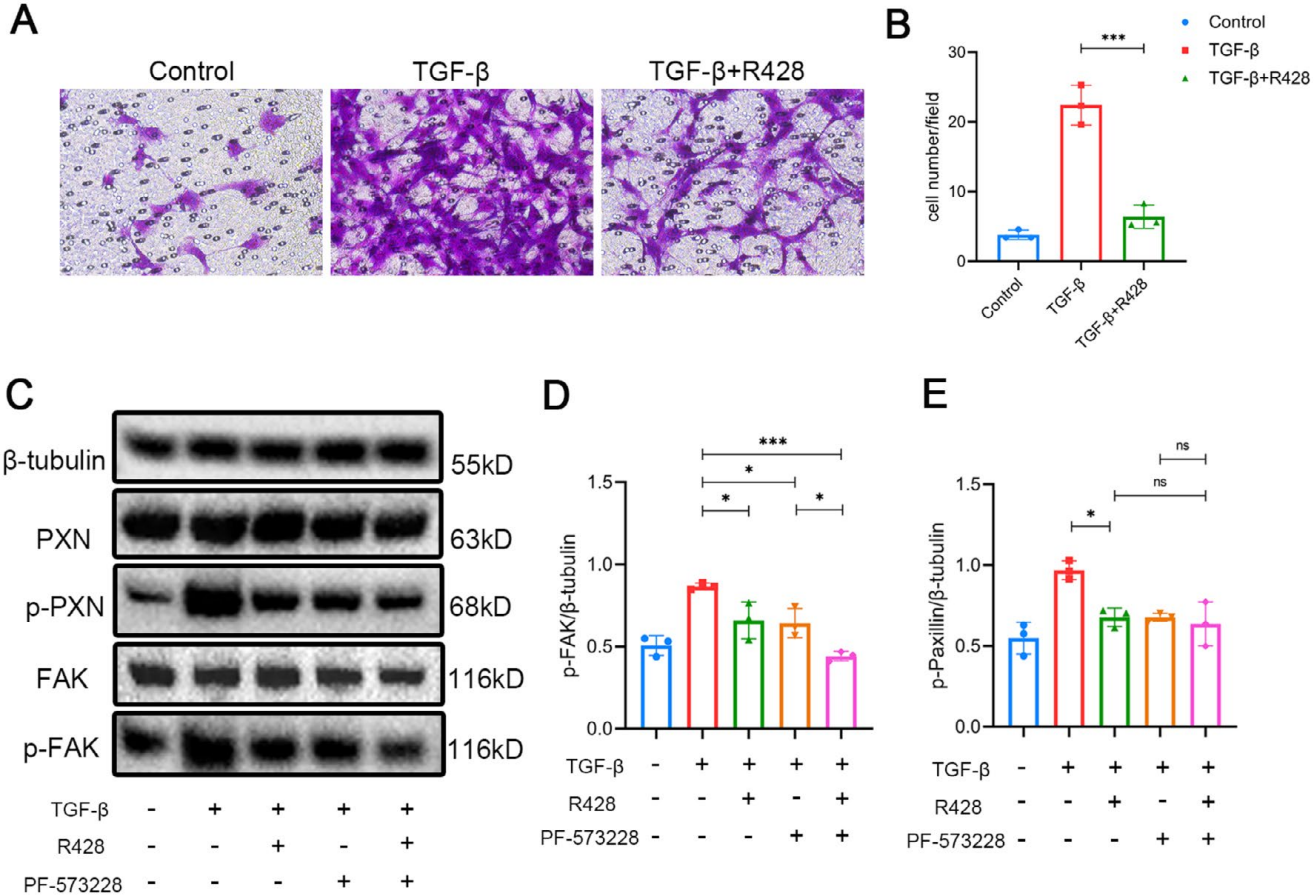
Inhibition of Axl or FAK can reduce the activation of PXN in fibroblasts. The extracted primary fibroblasts were treated with inhibitors for 4 h, then TGF‐β or PBS were added and follow‐up experiments were performed 48 h later. The concentration of R428 was 2 mmol/L, and the concentration of PF‐573228 was 3 mmol/L. (A) Photograph of the cell invasion experiment described above (original magnification: ×200). (B) The invasion experiment of the above cells was quantitative, and five random visual fields were selected from each Transwell under ×400 magnification for statistics. (C) Cell proteins were extracted and the protein levels of β‐tubulin, PXN, p‐PXN, FAK and p‐FAK were assessed by western blotting. (D) Protein quantification of p‐FAK in Figure C. (E) Protein quantification of p‐paxillin in Figure C. *n* = 3 mice/group, */***p < 0.05/0.001.

Focal adhesion kinase (FAK) plays an important role in cell migration and adhesion. It may phosphorylate PXN to facilitate FA turnover [20]. Therefore, we speculate that Axl may affect PXN phosphorylation by activating FAK, ultimately promoting FA turnover. We extracted primary fibroblasts from wild‐type mice and reproduced the same phenotype as Ax1^−/−^ mice after in vitro administration of R428 (Figure 9A,B). We found that Axl inhibitors can lead to a decrease in FAK phosphorylation levels, which is more pronounced when combined with FAK inhibitors, indicating that Axl may partially regulate FAK activation (Figure 9C,D). Next, we demonstrated that TGF‐β can significantly induce PXN phosphorylation. FAK inhibitor PF‐573228 or Axl inhibitor can inhibit TGF‐β‐induced PXN phosphorylation, and there was no statistically significant difference in p‐PXN levels between the two drug combination groups and the monotherapy group (Figure 9C,E), indicating that Axl may partially regulate fibroblast FA by promoting FAK phosphorylation.

### 2.8 Plasma Axl May Act as a Biomarker for IPF

**FIGURE 10:**
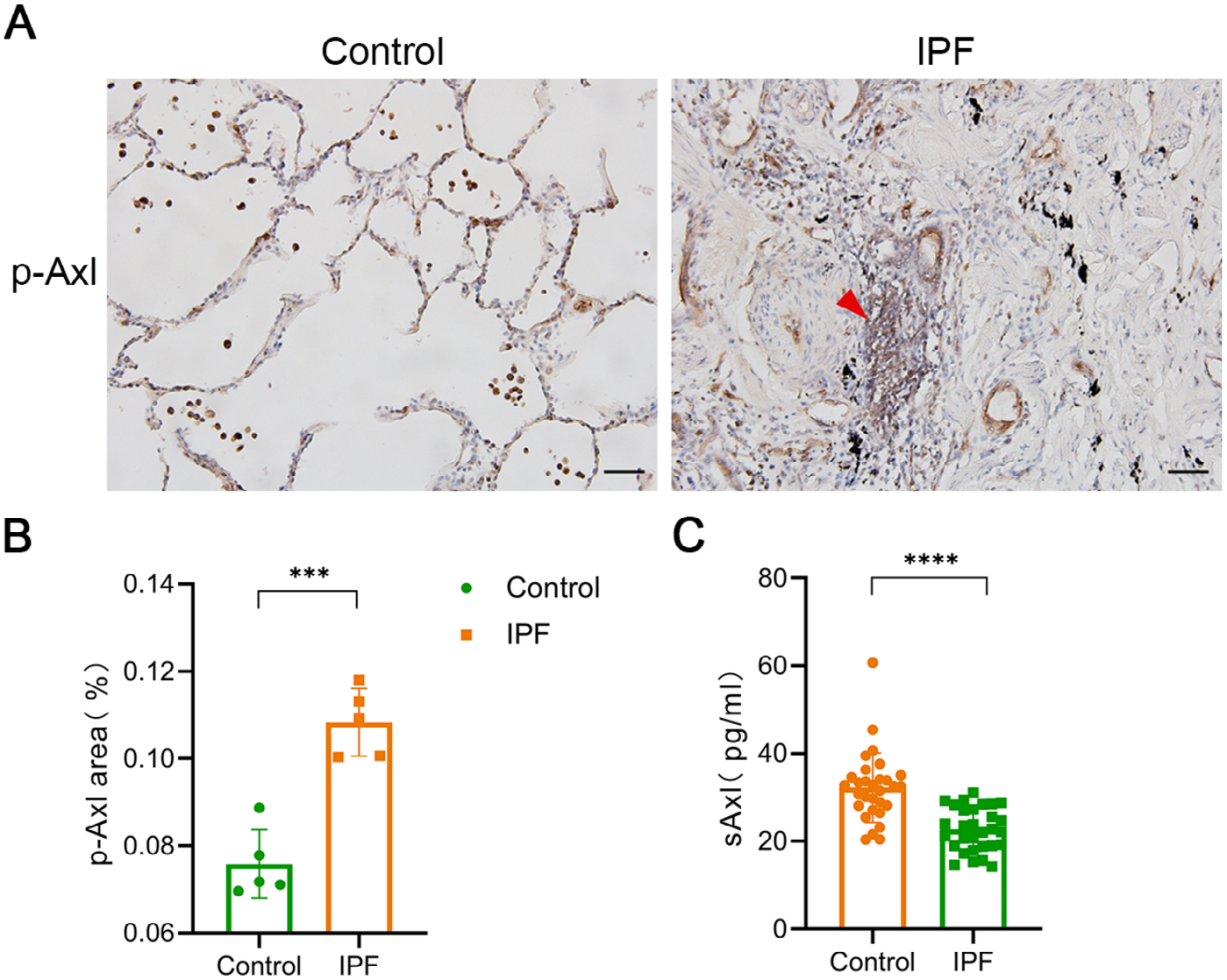
Changes in p‐Axl and s‐Axl in lung tissues and blood of patients with IPF. (A) Immunohistochemistry of lung tissues in healthy individuals and patients with IPF. Scale bars: All 20 μm (*n* = 5 people/group). (B) Immunohistochemical quantitative statistics in Figure A. Five visual fields were randomly selected for each slide under a × 400 magnification, and Image J was used for quantitative analysis. (C) SAxl levels in plasma of healthy people and IPF patients (*n* = 30 people/group). ***/****p < 0.001/0.0001.

To verify the Axl pathway activation in IPF patients, we detected the phosphorylated Axl (p‐Axl) as the activated form of Axl by IHC of paraffin sections from human lung tissues. Data showed that a small number of p‐Axl‐positive cells were found in the alveoli of healthy controls. Scar formation and honeycomb changes could be observed in the lungs of IPF patients, and a large number of p‐Axl‐positive cells can be found in areas with severe fibrotic lesions (Figure 10A,B).

Since soluble Axl (sAxl) may reflect the status of Axl pathway activation in the tissues, we tested plasma sAxl level to be a potential biomarker for IPF patients. We collected plasma from 30 healthy adults and 30 IPF patients with comparable baseline characteristics (Table 1). ELISA detection of sAxl showed that sAxl in plasma from IPF patients was significantly lower than that in the control group (Figure 10C), suggesting that sAxl may serve as a plasma marker for the diagnosis of IPF.

**TABLE 1:** Gender and age differences of patients.

	Control group (*n* = 30)	IPF group (*n* = 30)	*p*
Age (years)	57.97 ± 4.47	60.10 ± 5.00	0.0867
Gender (number, %)			
Male	22 (73.33)	25 (83.33)	0.3472
Female	8 (26.67)	5 (16.67)	

*Note:* Data are expressed as mean ± standard deviation or count (percentage). *p* > 0.05, indicating no statistical difference.

## 3 Discussion

In this study, we investigated the role of Axl in macrophages and fibroblasts and its temporal impact on pulmonary fibrosis. We also explored the potential of plasma Axl to be a biomarker for IPF.

Axl is an important regulatory molecule in the inflammatory response of macrophages, and its functional regulation of macrophages may be achieved by affecting their polarisation. Literature shows that after myocardial infarction, Axl promotes macrophage glycolysis metabolism and inflammatory factor production, exacerbates ventricular remodelling and leads to heart failure [21]. Research on tumour‐related macrophages also shows that TAM molecules promote macrophage polarisation towards M2 [22]. However, the effect of Axl on macrophage polarisation in the process of pulmonary fibrosis is unclear. Our research has shown that in the acute phase of lung injury, Axl inhibition mainly promotes M1‐type polarisation of macrophages, inhibits M2‐type polarisation and produces proinflammatory effects.

Our unpublished data based on single‐cell sequencing analysis showed that due to the significant reduction in tissue‐resident AMs on Day 3 after BLM stimulation, the AM pool was mainly supplemented by MoMs. The number of monocytes peaked in lung tissue on Day 3 after BLM stimulation, while the number of newly transformed AMs steadily increased from Day 0 to Day 7. Therefore, in this study, we further investigated the effect of Axl on the polarisation of MoMs. We used BMDMs for in vitro polarisation experiments. Correspondingly, we used Cx3cr1‐Cre mice to mediate Axl knockout in the monocytes/macrophages and investigate its impact on pulmonary fibrosis. In the process of fibrosis, M2 macrophages are considered to be the cell type that promotes fibrosis. However, our study showed that although Axl played a role in promoting M2 polarisation, the knockout of Axl in the monocytes/macrophages ultimately led to the exacerbation of pulmonary fibrosis, which may be caused by the more pronounced inflammatory responses, as demonstrated by the application of Axl inhibitors in the acute phase. Therefore, our result implies a risk of using Axl inhibitors during the acute or acute exacerbating stages of pulmonary fibrosis.

By preventing the activation of the NLRP3 inflammasome, Gas6‐activated Axl promotes the autophagy of macrophages in acute hepatic injury, thereby reducing the inflammatory response in the liver [23]. It was discovered in a study by Jie Zhang et al. that the NLRP3 inflammasome can promote M1‐type polarisation of macrophages [24]. Additionally, various studies have discovered that the inflammasome‐related pathways are inhibited when macrophages are polarised towards the M2 type [25, 26, 27]. The promotion of the activation of the NLRP3 inflammasome may be the mechanism by which inhibition of Axl induces macrophage polarisation to the M1 type and prevents it from happening to the M2 type; this is one area of future research to be explored.

Subsequently, we found that the inhibition of Axl during fibrosis phase could reduce pulmonary fibrosis. The role of Axl on fibroblasts has been linked to an aggressive phenotype as demonstrated by experiments using its inhibitor R428 [16]. In our study, we confirmed that Axl could promote the proliferation, invasion and collagen secretion of fibroblasts induced by TGF‐β by using primary Axl^−/−^ fibroblasts. Specifically, the Axl‐FAK‐PXN axis may accelerate the FA turnover that facilitates the migration of the myofibroblasts.

The Axl‐specific small‐molecule inhibitor R428 has entered the stage of clinical trials for tumour treatment, and the completed trials have demonstrated its potential as a clinical drug [28]. ONO‐7475 is also undergoing clinical trials in Japan. The high selectivity of these two inhibitors makes them have strong potential for clinical application. Axl mainly functions through PI3K/AKT/mTOR, JAK/STAT, NF‐κB and RAS/RAF/MEK/ERK signalling pathways to facilitate tumour cell survival, antiapoptosis signalling, mitogenesis, migration and invasion [29]. In addition, Axl activation may trigger actin remodelling via Rac1 and affect FA turnover and cell spreading which leads to tumour cell migration and invasion [30]. However, the mechanism that underlines the function of Axl to promote myofibroblast migration and invasion in pulmonary fibrosis is not clear. Here, we demonstrated that upon Axl knockout, the number of FA significantly increased and the FA area significantly enlarged, indicating a decreased FA turnover rate which may lead to the inhibition of fibroblasts movement and pulmonary fibrosis. FAK plays an important role in FA regulation. Although the Axl/FAK/NF‐κB signalling pathway has been reported [31], the regulation mechanism of Axl on PXN is not clear in myofibroblasts. We further demonstrated that Axl may phosphorylate FAK, which then activates PXN and promotes FA turnover.

Axl can be cleaved by metalloproteinases to produce sAxl. The level of sAxl may be related to Axl phosphorylation in cells. However, their relationship is not fully understood. Axl and its phosphorylation level paralleled hepatic stellate cell activation, and Axl serum levels increased in alcoholic liver disease and hepatitis C virus‐infected patients [32]. In contrast, sAxl may negatively regulate the Axl pathway activation by acting as a decoy receptor to block the binding of Gas6 to the membrane receptors [33]. The sAxl levels in the cell culture supernatant of primary fibroblasts extracted from lung tissue of IPF patients were significantly lower than those in the control group [16]. The plasma sAxl levels in patients with abdominal aortic aneurysms were lower than those in the control group, while the serum sAxl levels in patients with larger aneurysms were lower [34].

To investigate the relationship between plasma sAxl levels and activation of the Axl pathway in lung tissue, and to explore whether sAxl can serve as an IPF biomarker, we first verified that the Axl phosphorylation level was higher in the lung tissues of IPF patients. This result is consistent with the previous report [16]. In addition, increased Axl phosphorylation has also been reported in liver fibrosis [32] and tumour tissues [35]. We then discovered that plasma sAxl levels in patients with IPF were significantly lower than those in the control group. Our result implied that the lower level of sAxl in IPF patients may lead to an attenuated suppression of Gas6 signal, resulting in increased Axl pathway activation in other cells in lung tissue such as fibroblasts and macrophages. Due to the limitation of small number of patients in our study, further validation is needed to be done in larger cohorts. In addition, as Axl plays a role in the pathophysiological processes of other organs in the body, the specificity of sAxl as a biomarker for IPF still needs to be evaluated.

**FIGURE 11:**
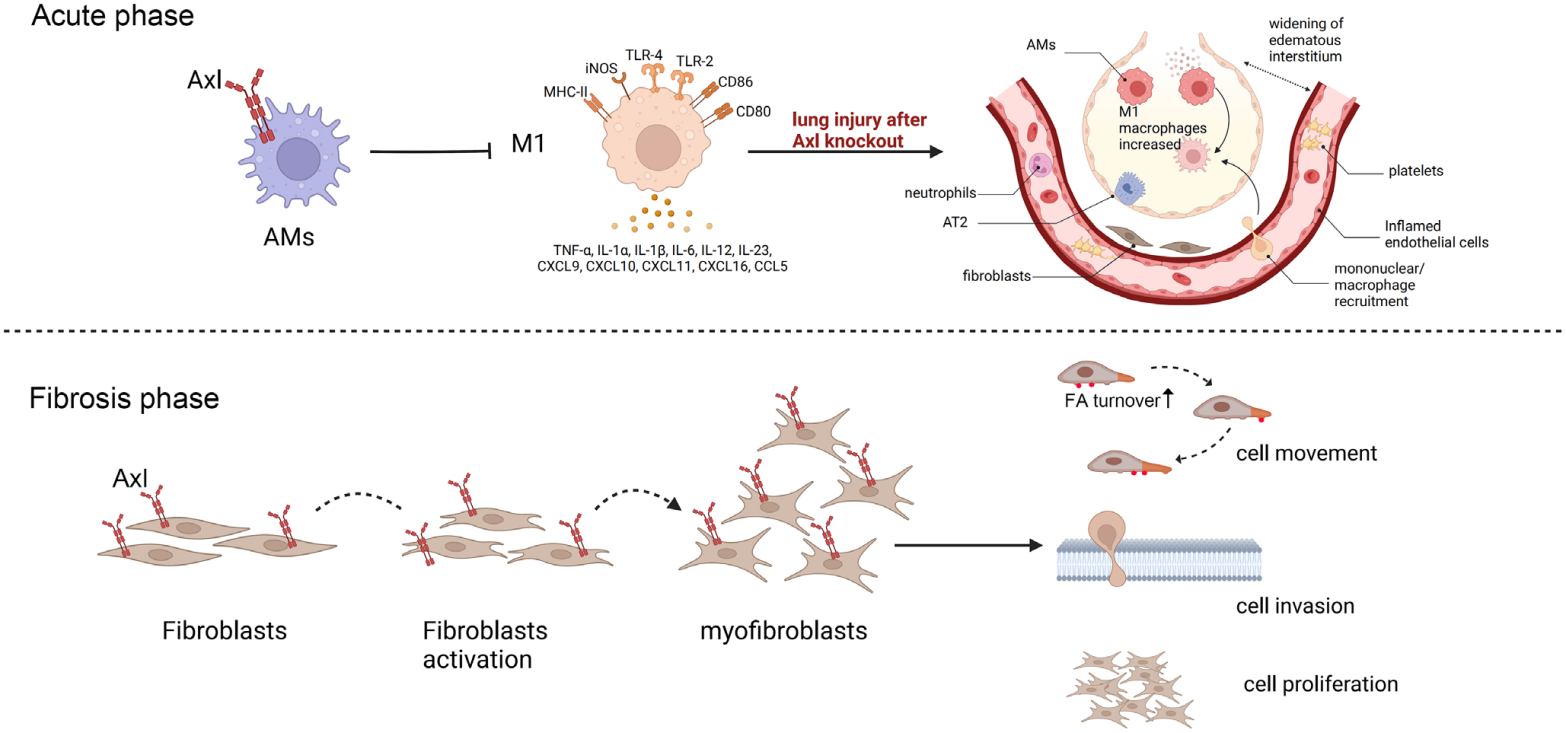
The role of Axl varies in different cells of lung tissue. In the acute phase of pulmonary fibrosis, inhibiting Axl promotes macrophage polarisation towards M1 type, thereby exacerbating the inflammatory response. During the fibrosis phase, Axl promotes the activation of fibroblasts by facilitating their migration, invasion and proliferation.

Taken together, we found that Axl has an opposite dual effect in BLM‐induced pulmonary fibrosis in mice. In the period of acute lung injury, Axl activation can inhibit macrophage M1 polarisation and reduce the release of inflammatory factors, thus alleviating lung tissue injury. In the fibrosis stage, Axl can promote the activation of fibroblasts, thus aggravating the progression of lung fibrosis, as shown in Figure 11. In fact, the opposite effect of Axl‐related pathways has been reported in the acute‐to‐chronic progression of liver diseases [36], but our study is the first to describe this phenomenon in pulmonary fibrosis. Our findings provide an important rationale for the future treatment of pulmonary fibrosis targeting Axl in different stages of disease development, which may improve the therapeutic efficacy and outcomes.

## 4 Methods

### 4.1 Human Samples

Five lung tissues from patients with IPF and five lung transplant donors were collected from China‐Japan Friendship Hospital. Plasma was obtained from 30 patients with IPF patients and 30 healthy adults. There were no statistical differences in the age and sex of the patients (Table 1). The diagnosis of IPF was based on guidelines presented by the American Thoracic Society, the European Respiratory Society, the Japanese Respiratory Society and the Latin American Thoracic Society. The study was approved by the Ethics Committee of China‐Japan Friendship Hospital (2021‐99‐K60).

### 4.2 Animal Models and Breeding

Wild‐type mice were purchased from Charles River, and Axl systemic knockout mice and conditional knockout mice were purchased from Cyagen Inc. All mouse strains were C57BL/6 [37]. For systemic knockout mice, Axl^+/−^ and Axl^−/−^ were combined by cage breeding. Mouse DNA was extracted using the TaKaRa MiniBEST Universal Genomic DNA Extraction Kit, amplified by a PCR instrument and subjected to electrophoresis in 2% agarose gel. Axl^−/−^ mice were compared with Axl^+/−^ mice in the same cage.

For conditional knockout mice, Axl^fl/fl^ mice were bred with Cx3cr1‐Cre::Axl^fl/fl^ mice and Col1a2‐Cre::Axl^fl/fl^ mice in the same cage, respectively, using the same cage Axl^fl/fl^ mice as the control.

All experimental mice were male mice aged 6–8 weeks, with age differences of less than 2 weeks. They were housed in 12 h/12 h light/dark alternating cycles with controlled temperature (21°C–25°C) and adequate food and water. The experiments were approved by the Experimental Animal Ethics Committee of the China‐Japan Friendship Hospital (Zryhyy61‐20‐10‐1).

### 4.3 Mouse Models of Pulmonary Fibrosis

Wild‐type mice or Axl^+/−^ and Axl^−/−^ male healthy mice of 6–8 weeks old were randomly assigned to gas anaesthesia with isoflurane (3%), and then fixed on a scaffold. Endotracheal intubation was performed with mouse laryngoscope. After a successful hose test, BLM (2.0 mg/kg, Hisun Pfizer Pharmaceuticals Co, Hangzhou, China) was added and the control group was treated with the same dose of normal saline. Wild‐type inhibitor groups received intragastric administration of R428 [16, 38] (5 mg/kg, MedChem Express, Monmouth Junction, NJ, USA), the inhibitor of Axl or ONO‐7475 [39] (10 mg/kg, Selleck Chemicals, Houston, TX, USA), the inhibitor of both Axl and Mer, respectively, while the blank control group and the BLM group received the same dose of sodium carboxymethylcellulose (CMC‐Na, Solarbio, Beijing, China) solution. For Axl^fl/fl^ mice, Cx3cr1‐Cre::Axl^fl/fl^ mice and Col1a2‐Cre::Axl^fl/fl^ mice were injected intraperitoneally with tamoxifen (40 mg/kg, Sigma‐Aldrich, St. Louis, MO, USA) for 5 days prior to modelling, and BLM administration was started after 1 week. After modelling, materials are collected according to the days required by the experiment. Mice were anaesthetised with an intraperitoneal injection of 2% pentobarbital and lung tissues were washed with phosphate‐buffered saline (PBS, Solarbio, Beijing, China).

### 4.4 Histopathological Staining

Lung tissues were immersed in 4% paraformaldehyde solution for 24 h, then sliced after paraffin embedding (Slice thickness for 5 μm), baked in an oven at 65°C for 2 h, dewaxed with xylene and rehydrated, and then stained with H&E and Masson, respectively. To scan the slices, they were put in a slice scanner. Regarding the lung tissues obtained on Day 21, scoring was done in a single‐blind fashion in accordance with Ashcroft et al.'s recommendation [40]. Regarding the lung tissues obtained on Day 3 or day 7, scoring was done in a single‐blind fashion in accordance with the Official American Thoracic Society Workshop Report [41].

For IHC, a generic SP kit (ZSBg‐BIO, Beijing, China) was used for sealing and colour development. Phospho‐Axl antibody (1:50, Invitrogen, Carlsbad, CA, USA), anti‐α‐SMA antibody (1:2000, Abcam, Cambridge, MA, USA), Col‐1 antibody (1:1000, Cohesion Bioscience, London, UK), antimyeloperoxidase rabbit antibody (1:500, Servicebio, Wuhan, China) and goat anti‐rabbit IgG H&L (1:3000, Abcam, Cambridge, MA, USA) were used. Under a 40× microscope, five randomly chosen fields were examined for each slide and Image J software was then used for analysis.

### 4.5 Primary BMDM Culture

Healthy Axl^+/−^ and Axl^−/−^ mice of 6–8 weeks of appropriate ages were obtained and euthanised. After neck removal, the mice were soaked in 75% alcohol for 5 min. The skin, fascia and muscle of the lower extremities of the mice were carefully peeled with ophthalmic scissors, and the intact femur and tibia were retained and transferred to a sterile table. The bones were soaked in 75% alcohol for 1 min and washed three times with sterile PBS. The middle part of the bone was removed from both ends of the bone, and the bone marrow cavity was rinsed with sterile PBS with a 1‐mL syringe. The suspension was screened through 70 μm cell sieve, cleaved, washed and finally, the bone marrow was collected in DMEM medium containing 10% foetal bovine serum (FBS). The cell suspension was collected after incubation overnight as bone marrow–derived mononuclear cells and stimulated with 20 ng/mL of macrophage colony‐stimulating factor (M‐CSF, Peprotech, Rocky Hill, NJ, USA) for 7 days. Macrophage phenotypes were identified by flow cytometry F4/80^+^CD11b^+^. M1 macrophages were induced to polarise by adding 100 ng/mL lipopolysaccharide (LPS, Sigma‐Aldrich, St. Louis, MO, USA) and 20 ng/mL interferon‐γ (IFN‐γ, Peprotech, Rocky Hill, NJ, USA) for 24–48 h. M2 macrophages were induced to polarise by adding 20 ng/mL interleukin‐4 (IL‐4, Peprotech, Rocky Hill, NJ, USA) for 24–48 h.

### 4.6 Primary Fibroblasts Culture

Healthy Axl^+/−^ and Axl^−/−^ mice at 6–8 weeks of age were euthanised as indicated above and were then soaked in 75% alcohol. Lung tissues were exposed with sterile surgical instruments, and lung tissues were perfused with normal sterile saline. The tissue fragments were evenly spread in culture bottles using the tissue adhesion method. After 2 h of dry adhesion, DMEM medium containing 20% FBS was added. After 7–9 days of culture, DMEM medium containing 10% FBS was purified by the differential adhesion method. The cells used in this study were Generations 3–5 and were identified by immunofluorescence using vimentin and α‐SMA (Figure S2). Antibody information: antivimentin antibody (1 μg/mL, Abcam, Cambridge, MA, USA) and anti‐α‐SMA antibody (1:300, Abcam, Cambridge, MA, USA).

### 4.7 Single‐Cell Suspension Preparation

Single‐cell lung tissue suspensions were prepared using the lung dissociation kit (Miltenyi Biotec GmbH, Bergisch Gladbach, Germany). Briefly, the unilateral lung tissue removed was placed in tissue storage solution (Miltenyi Biotec GmbH, Bergisch Gladbach, Germany), and the tissue, digestive enzymes and buffer solution were placed in a homogenate tube in proportion according to the instructions for moderate grinding and then digested in a 37°C water bath. The mixture in the homogenate tube was filtered through a 70 μm cell filter, the filtrate was collected and then rinsed with cracked red and PBS for follow‐up experiments. For the preparation of single‐cell suspensions of cultured cells, we digested macrophages using ACCUTASETM cell digestive solution (Yeasen, Jiangsu, China) and then washed cells with PBS.

### 4.8 Flow Cytometry

Single‐cell suspensions were prepared; the number of cells per tube was 1 × 10^6^/100 μL. The zombie UVTM fixable viability kit (Biolegend, San Diego, CA, USA) was used to detect cell viability with incubation for 20 min at room temperature without light. Cell suspension staining was performed after washing with cell staining buffer (Biolegend, San Diego, CA, USA) and cells were then sealed with truStain FcX plus (anti‐mouse CD16/32) antibody (Biolegend, San Diego, CA, USA). The primary antibody was then added to the fixation buffer (Invitrogen, Carlsbad, CA, USA) and incubated for 20 min. After washing, the suspensions were placed in IC fixation buffer (Invitrogen, Carlsbad, CA, USA) for 30 min. After washing with intracellular staining permeabilisation wash buffer (Biolegend, San Diego, CA, USA), the primary antibody was added and allowed to incubate for 20 min in the dark. After washing two times, Beckman Coulter (CytoFLEX LX) was used for analysis. All primary antibodies used were purchased from Biolegend (San Diego, CA, USA) as follows: CD45‐BV510, F4/80‐BV510, CD11b‐BV650, LY6C‐BV785, MHC II‐FITC, LY6G‐PE, CD64‐PE CY7, Siglec F‐AF647, CD86‐AF700, CD3E‐APC CY7, CD19‐APC CY7, NK1.1‐APC CY7 and CD206‐Percp Cy5.5.

### 4.9 Cell Invasion Assay

After primary fibroblast serum starvation for 12–24 h, the medium was changed and 5 ng/mL TGF‐β (MedChem Express, Monmouth Junction, NJ, USA) was added for 48 h before cell layers were digested with 0.25% trypsin (Gibco, Grand Island, NY, USA). Experiments involving inhibitors were treated with inhibitors for 4 h prior to the addition of TGF‐β. Cells were seeded at a density of 2 × 10^4^ cells/well in Transwell plates (Corning Costar, New York, USA) lined with a matrix layer (Yeasen, Jiangsu, China). Serum‐free DMEM medium was added to the upper part of the chamber, DMEM medium containing 10% FBS was added to the lower part of the chamber and the cells were cultured in the incubator for 20 h. The upper part of the medium and matrix layer was removed and stained with crystal violet. The cells were observed under a 40× microscope using an Olympus (BX51), and five randomly different fields of view were recorded for each specimen.

### 4.10 Cell Proliferation Experiment

Cell proliferation capacity was examined using CCK‐8 and EDU assays. For the CCK‐8 assay: primary fibroblasts were distributed into 96‐well plates with 8 × 10^3^ cells per well. After attaching the cells to the wall, the cells were serum starved for 12–24 h, and then fresh medium with 5 ng/mL TGF‐β was added for 48 h. According to the instructions of the cell counting kit (Dojindo Laboratories, Kumamoto, Japan), readings at the wavelength of 450 nm were obtained using a multifunctional enzyme label instrument (Tecan, Spark, North Carolina, USA). For the EdU assay, the BeyoClick EDU‐647 cell proliferation detection kit (Beyotime, Shanghai, China) was used. The cells were inoculated in the 24‐well plate at a density of 1 × 10^4^ cells/well. After the same stimulation with TGF‐β, a click reaction solution was added according to the instructions, incubated at room temperature in the dark for 30 min and then washed in TBST three times. Finally, antifluorine‐quenched DAPI Fluoromount‐G (Yeasen, Jiangsu, China) was added and slides were observed under a 40× laser confocal microscope (Zeiss, LSM800, Jena, Germany) with five random fields recorded for each sample.

### 4.11 Immunofluorescence

Primary fibroblasts were seeded in 24‐well dishes at a density of 2 × 10^4^ cells/well, and the plate was removed after the cells had attached to the wall. After fixation, permeability and sealing, the primary antibody was incubated overnight at 4°C overnight, followed by incubation with the secondary antibody. Slides were sealed with DAPI and photographed under a 40× laser confocal microscope.

### 4.12 Western Blotting

Lung tissues and cultured cells were cleaved using high‐potency RIPA tissue/cell lysis solution (Solarbio, Beijing, China), and preserved at −80°C with protease inhibitors, phosphatase inhibitors and protein loading buffers (all purchased from Solarbio). Electrophoresis experiments were performed in 4%–15% BeyoGel Plus PAGE prefabricated glue (Beyotime, Shanghai, China). After transferring the film, they were sealed in a QuickBlock Western sealing solution (Beyotime, Shanghai, China) for 30 min. The primary antibody was incubated at 4°C overnight and the secondary antibody was incubated at room temperature for 2 h. Finally, the Bio‐Rad gel imager (Bio‐Rad, Hercules, CA, USA) was used for development analysis. Bands were quantified using Image J software (National Institutes of Health, Bethesda, MD, USA). Reagents used for the electrophoresis and transmembrane studies were purchased from Beyotime (Shanghai, China). Primary antibodies used include anti‐β‐tubulin antibody (1:1000, Boaoruijing, Beijing, China), anticollagen I antibody (1:500, Cohesion Bioscience, London, UK), anti‐FAK antibody (1:300, Bioss, Beijing, China), anti‐phospho‐FAK (1:300, Bioss, Beijing, China), anti‐PXN (1:1000, Bioss, Beijing, China), anti‐phospho‐PXN (1:1000, Bioss, Beijing, China) and anti‐α‐SMA (1:20,000, Abcam, Cambridge, MA, USA). Secondary antibodies used were goat anti‐rabbit IgG H&L (1:5000, Abcam, Cambridge, MA, USA), goat anti‐mouse IgG H&L (1:5000, Abcam, Cambridge, MA, USA) and donkey anti‐goat IgG H&L (1:3000, Abcam, Cambridge, MA, USA).

### 4.13 Enzyme‐Linked Immunosorbent Assay

The DuoSet ELISA kit (R&D Systems, Minneapolis, MN, USA) and the DuoSet ancillary reagent kit (R&D Systems, Minneapolis, MN, USA) were used to determine sAxl concentration in human plasma. Sample preparation: The supernatant was utilised to make plasma after the whole blood was centrifuged for 5 min at 2000 rpm and then for 10 min at 13,200 rpm. The plasma was kept for an extended period of time at −80°C. Plasma was diluted to 1:75 before measurements. Specific steps were conducted according to the manufacturer's instructions. ELISACalc software was utilised to create standard curves following the acquisition of data from the microplate reader, in order to compute the ultimate outcomes.

### 4.14 RNA Sequencing (RNA‐Seq)

Wild‐type mice were selected and the inhibitor group were given R428 or ONO‐7475 after BLM modelling, lung tissue was taken on Day 3 and Day 7 and sent to Gene Denovo for RNA sequencing. A single‐sample gene aggregate analysis (ssGSEA) was used to calculate a marker set of genes based on the ‘GSVA’ R package (v.1.46.0) [42]. CIBERSORT is a cellular biomarker RNA mixture analysis tool based on a set of 22 immune cell subtypes of gene expression characteristics. Calculate the macrophageal cell subtype scores of mice by using sound code downloaded from the official CIBersORT website [43].

### 4.15 Statistical Analysis

At least three biological replicates were performed for all experiments. Data were sorted using the WPS Office 2021 PC version (Beijing, China), and statistical analysis was performed using GraphPad Prism 8 (Version X; La Jolla, CA, USA). The *t* test or the nonparametric test was used for the comparison of two groups, and the one‐way analysis of variance or the nonparametric test was used for the comparison of multiple groups. *p* < 0.05 was considered statistically significant.

## Author Contributions

**Xinyu Zhao:** data curation (equal), investigation (equal), methodology (equal), validation (equal), writing – original draft (equal). **Yupeng Li:** methodology (equal), software (equal), validation (equal). **Shengnan Yang:** conceptualization (equal), formal analysis (equal). **Yicong Chen:** formal analysis (equal). **Kaiwei Wu:** visualization (equal). **Jing Geng:** software (equal). **Peipei Liu:** resources (equal). **Zai Wang:** supervision (equal), writing – review and editing (equal). **Huaping Dai:** funding acquisition (equal), project administration (equal). **Chen Wang:** funding acquisition (equal), project administration (equal).

## Ethics Statement

The study was approved by the Ethics Committee (2021‐99‐K60) and the Experimental Animal Ethics Committee of China‐Japan Friendship Hospital (Zryhyy61‐20‐10‐1).

## Consent

We acquired written informed consent from each participant for publication.

## Conflicts of Interest

The authors declare no conflicts of interest.

## Supporting information


Data S1.

## Data Availability

The experimental data presented in this study are included in the article/Supporting Information. Further inquiries can be directed to the corresponding authors. RNA sequencing data have been uploaded to the NCBI SRA (https://www.ncbi.nlm.nih.gov/sra), and the SRA data are PRJNA1023631.
