# Supplementary material for: Orderly Regulation of Macrophages and Fibroblasts by Axl in Bleomycin‐Induced Pulmonary Fibrosis in Mice
Source: J Cell Mol Med. 2025 Jan 8;29(1):e70321. doi: 10.1111/jcmm.70321 (PMC11710931; doi:10.1111/jcmm.70321)
Supplement: Supplementary file 1 — Data S1. [file JCMM-29-e70321-s001.docx]

**Supplementary Files**


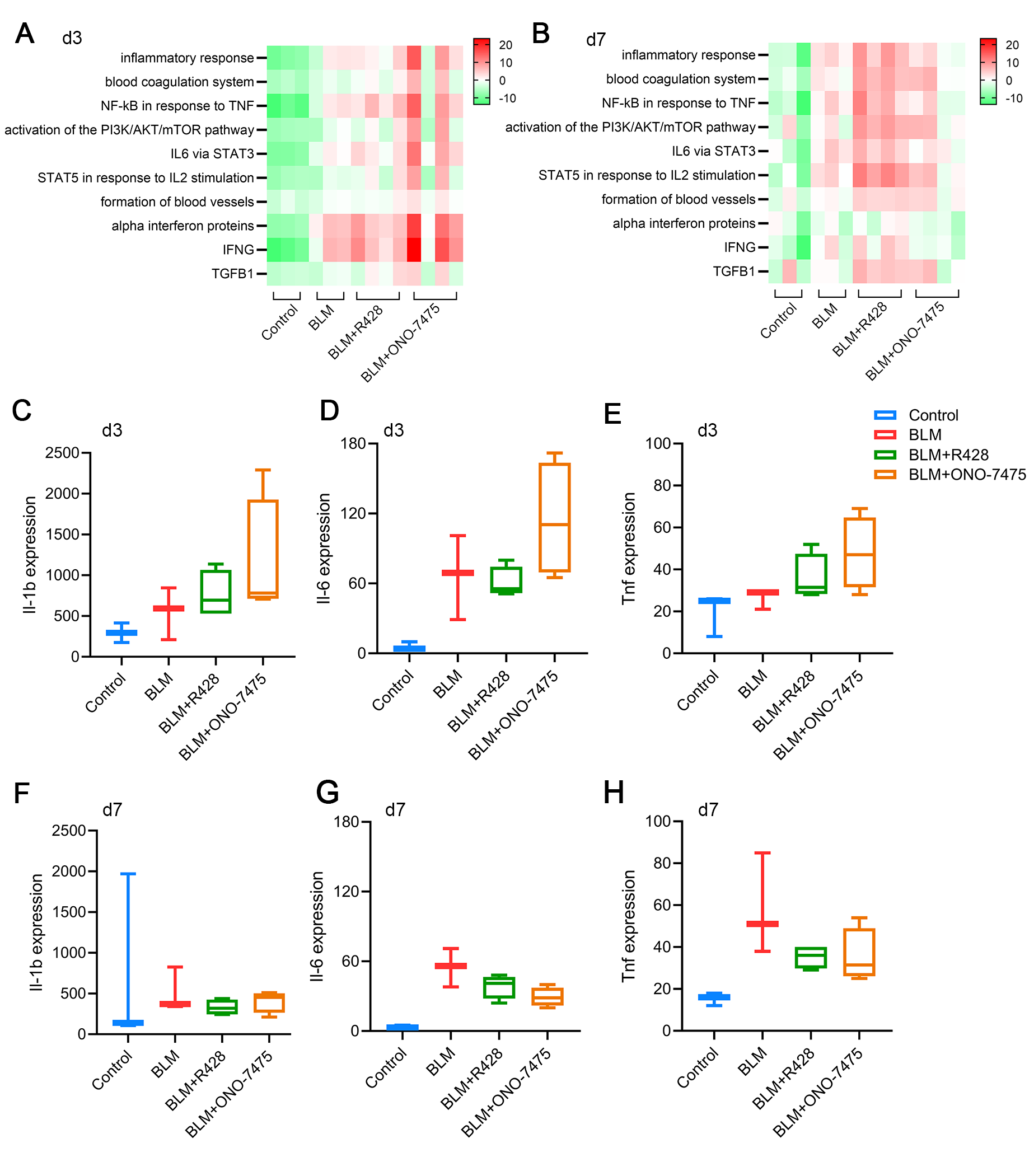


**Figure S1**

**RNA sequencing results of lung tissues that inhibit Axl during acute inflammatory response.** (A-B) ssGSEA analysis. (C-E) Expression levels of Il-1b (C), Il-6 (D) and Tnf (E) by transcriptome sequencing in lung tissues of mice on day 3. (F-H) Expression levels of Il-1b (F), Il-6 (G) and Tnf (H) by transcriptome sequencing in lung tissues of mice on day 7. n=3-4 mice/group, */**/***p < 0.05/0.01/0.001.


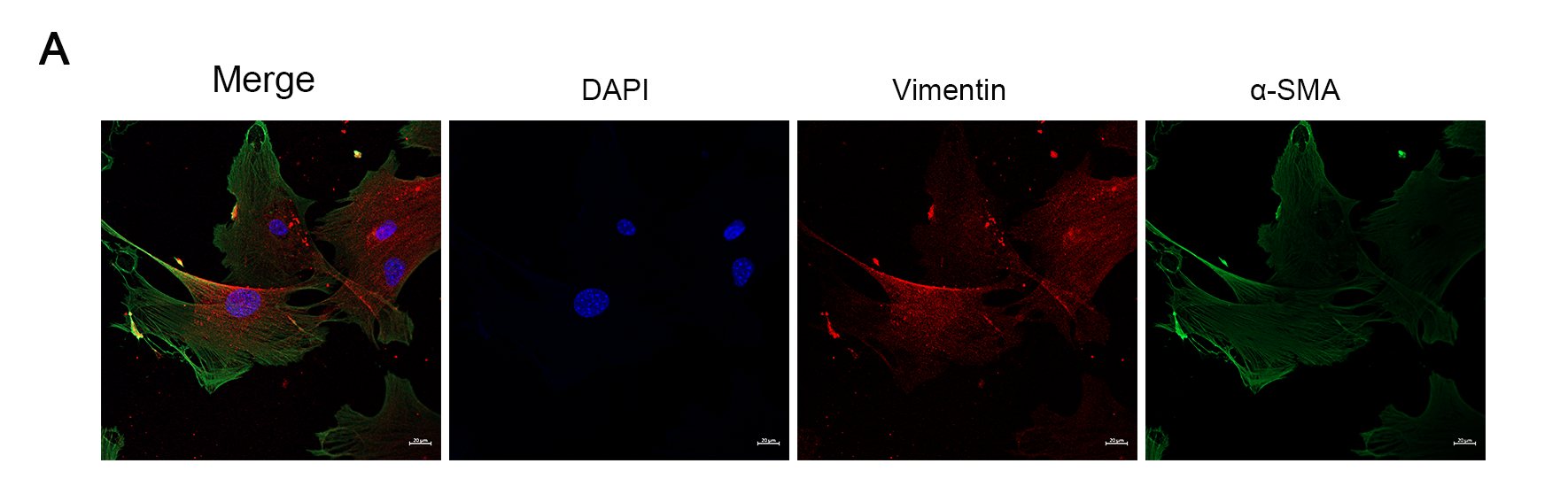


**Figure S2**

**Immunofluorescence identification of primary fibroblasts.** (A) Primary mouse fibroblasts were extracted by tissue adherence method, purified/isolated and cultured to the third generation, stained by immunofluorescence, and photographed under confocal laser microscope. Scale bars: 20 μm.
